# Scoping Review of Employer-Led Research Using Employee Health Claims Data

**DOI:** 10.1089/pop.2023.0140

**Published:** 2023-10-10

**Authors:** Naimisha Movva, Susan T. Pastula, Saumitra V. Rege, R. Jeffrey Lewis, Lauren C. Bylsma

**Affiliations:** ^1^ EpidStrategies, A Division of ToxStrategies, Rockville, Maryland, USA.; ^2^ ExxonMobil Biomedical Sciences, Inc. (EMBSI), Annandale, New Jersey, USA.; ^3^ Epidemiology Contractor (Retired EMBSI), Lavallette, New Jersey, USA.

**Keywords:** claims data, employee health plan, employer, scoping review, self-insured, workforce health

## Abstract

Employers may evaluate employee claims data for various reasons, including assessment of medical insurance and wellness plan efficacy, monitoring employee health trends, and identifying focus areas for wellness measures. The objective of this scoping review (ScR) is to describe the available literature reporting the use, applications, and outcomes of employee health claims data by self-insured employers. The ScR was conducted in a stepwise manner using an established framework: identifying the research question, identifying and selecting relevant studies, charting the data, and collating and reporting results. Literature searches were conducted in PubMed and Embase. Studies of self-insured employee populations that were conducted by the employer/s through May 2022 were identified using predefined criteria. Forty-one studies were included. The majority (90%) were cohort study designs; most employers (51%) were in industries such as aluminum production and health insurance providers. Twenty-four (59%) studies supplemented claims data with other sources such as human resource data to evaluate programs and/or health outcomes. A range of exposures (eg, chronic conditions, wellness program participation) and outcomes (eg, rates or costs of conditions, program effectiveness) were considered. Among the 25 studies that reported on patient confidentiality and privacy, 68% indicated institutional review board approval and 48% reported use of deidentified data. Many self-insured employers have used employee health claims data to gain insights into their employees' needs and health care utilization. These data can be used to identify potential improvements for wellness and other targeted programs to improve employee health and decrease absenteeism.

## Introduction

In 2020, 54.4% of the US population (over 177 million persons) had employer-based health insurance.^1^ Most large employers across industry sectors are self-insured and assume the financial risk of the employee health plans.^2,3^ For example, the 2020 Kaiser Family Foundation Survey of Employer Health Benefits reported that 67% of employed workers had health insurance coverage under self-insured arrangements.^4^

The relatively wide degree of health insurance coverage among the US workforce represents a potential data source to promote the health, well-being, and performance of employees. Improved employee health and well-being may not only benefit employees, but may also deliver a competitive business advantage for employers.^2,5^ Implementation of evidence-based approaches using employee medical claims data can potentially lead to improved health and productivity among the workforce while also reducing health care costs and absenteeism. As a result, there are numerous potential applications of employee health claims data to self-insured employers. These include employee health and wellness monitoring, determining potential health impacts of new technologies or safety measures, optimizing health plan efficacy, identifying health disparities, improving quality of care, and tailoring prevention/management programs to meet employee health needs. Claims data may also inform employers in identifying drivers of health care utilization over time and prioritizing specific areas to reduce costs; for example, increased claims for tobacco-related diseases may prompt employers to incentivize employees for taking measures toward smoking cessation. In light of the aforementioned applications, health claims data may prove an important tool for self-insured employers to develop and sustain an effective workforce.

Although a number of studies have been published recently that provide examples of employers evaluating their workforce health claims data,^2,5,6^ no scoping review (ScR) or systematic literature review has been conducted that has fully gauged the extent of the literature surrounding employer-led research of claims data. The objective of this ScR is to understand the ways in which self-insured employers have utilized or evaluated their employees' health claims data as well as the insights and benefits gained, and lessons learned from these analyses.

## Methods

A study protocol was developed and registered on Open Science Framework (https://osf.io/qup2g) on August 17, 2022, before initiating the ScR. The ScR was conducted in accordance with the framework proposed by Arksey and O'Malley and further refined by the Joanna Briggs Institute.^7,8^ The Preferred Reporting Items for Systematic Reviews and Meta-Analyses (PRISMA) guidelines—extension for scoping reviews (PRISMA-ScR)^9^ were followed in all aspects of the preparation, conduct, and reporting of this review.

### Eligibility criteria

As recommended by the Joanna Briggs Institute,^8^ the eligibility criteria used to determine relevant studies were organized by population, concept, and context. Studies of employee populations from self-insured employers were included. Relevant studies were required to describe how and why employee health care claims data were used to be included. Those utilizing only primary data collection methods, such as employee interviews or questionnaires, were not included.

Studies must have been initiated by the self-insured employers; studies analyzing employee populations sponsored by third parties were excluded. Interventional studies, clinical trials, and conference abstracts were not included. Studies published in languages other than English, conducted outside of the United States, or not meeting the population, concept, and context criteria were excluded from the ScR.

### Study identification, screening, and abstraction

An initial search of PubMed was conducted on July 18, 2022, to refine the final search string. Text words used in the title and abstract of the resulting articles were analyzed and relevant terms were used to build a comprehensive search that was run in PubMed and Embase on August 17, 2022. The final search strategy is provided in Table 1, with no date restrictions. DistillerSR software^10^ was utilized for study selection including deduplication, article screening, and abstraction, which resulted in a fully transparent and auditable process. Using the population, concept, and context criteria, 1 reviewer screened the titles and abstracts of the search hits.

**Table 1.** Literature Search Strategy

Database	PubMed	EMBASE
Terms for employer (#1)	“Employee”[TiAb] OR “employer”[TiAb] OR “company”[TiAb]	“employee”:ab OR “employee”:ti OR “employer”:ab OR “employer”:ti OR “company”:ab OR “company”:ti
Terms for self-insured (#2)	self-insured OR fully-insured OR “health plan” OR “claims data” OR “health claims” OR “medical claims” OR “employee health care” OR “employer burden” OR “workplace health” OR “workforce health”	“self insured” OR “fully insured” OR “health plan” OR “claims data” OR “health claims” OR “medical claims” OR “employee health care” OR “employer burden” OR “workplace health” OR “workforce health”
Filters	English	English; excluding “Conference Abstract,” “Conference Paper,” and “Conference Review”

The full text of the articles deemed relevant were examined independently by 2 reviewers. Studies included at the full text review stage were extracted in DistillerSR. Extracted data elements included general study information such as title and publication year, study characteristics including study design, location, and employer type, research objectives, study methodology, reported “exposures” (condition for entry into the study, eg, employment within a company, presence of a disease, or a workplace intervention) and “outcomes” (evaluated endpoints, eg, health care costs, prevalence of a condition, rate of workplace injury), measures to maintain confidentiality and patient privacy, strengths and limitations, and lessons learned.

Data extraction was completed by 1 reviewer and another reviewer performed an independent check of the data elements for accuracy. Disputes were resolved by a senior reviewer. Basic descriptive analyses (frequencies and percentages) were conducted on extracted study elements.

## Results

### Article identification

**FIG. 1.**
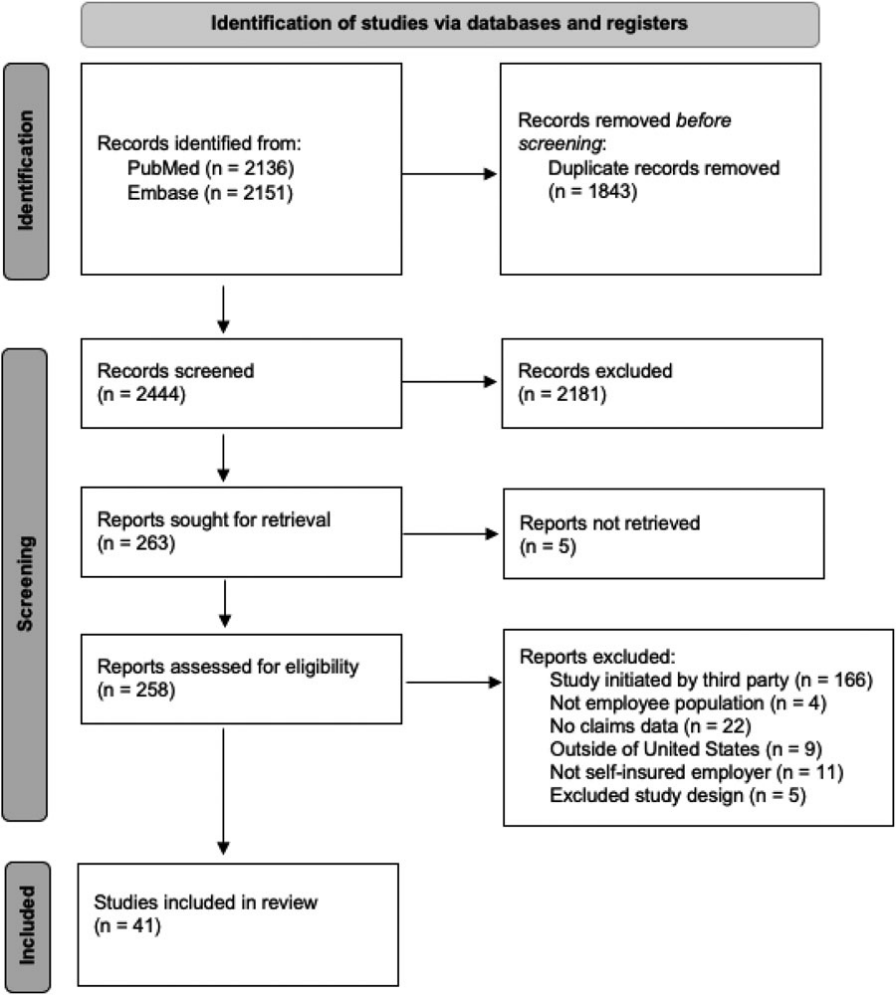
Study flow diagram.

Figure 1 shows the PRISMA flow diagram, which details the study inclusion at each stage. Searches yielded 4287 hits; 2444 hits were screened after deduplication at the title and abstract level. Screening of the abstracts against the study eligibility criteria resulted in 263 studies that were reviewed at the full-text stage. Among these, 222 studies were excluded at the full-text level: 166 studies were initiated by a third party, 22 studies did not contain claims data, 11 were not self-insured employers, 9 were conducted outside of the United States, 5 were excluded study designs, 4 were not employee populations, and the full-text PDF of 5 was unavailable. A total of 41 studies meeting the predefined eligibility criteria were thus included in the review.

### Study characteristics

Characteristics of the included studies are presented in Table 2. The 41 studies were published between 1985 and 2022. There were 2 (5%) cross-sectional, 1 (2%) case–control, 1 (2%) ecological, and 37 (90%) cohort studies. The majority of studies (*n* = 21; 51%) were conducted by industry employers such as aluminum production, information technology, and retail companies. Nine (22%) studies were conducted among university staff while 7 (17%) were conducted in hospital system employees.

**Table 2.** Characteristics of the Included Studies

Study	Study design	Time period	Employer	Employer category	Objective	Data sources	Exposure category*^a^*	Outcome category*^a^*	Patient privacy measures
Aguilar et al^14^	Cohort	2009–2011	Cerner	Industry	Assess the impact of an employer's onsite pharmacy on health plan members' medication adherence	Claims	Specific chronic condition: asthma, depression, diabetes, hyperlipidemia, hypertension	Medication adherence	IRB approval + deidentified data
Bernacki and Tsai^15^	Cohort	1992–2002	Johns Hopkins	University	Assess the success of integrated workers compensation claims management system	Claims + workers' compensation data	Employment	Workplace injury/illness	NR
Birnbaum et al^16^	Cohort	2004	Chevron Texaco	Industry	Assess the relative value of using laboratory, claims data to identify prevalence rates, and costs of metabolic syndrome	Claims + health screening, personnel data	Employment	Specific health condition rate/costs: metabolic syndrome	NR
Boscardin et al^17^	Cohort	2008–2009	Safeway	Industry	Examine the added value of self-reported health status data; predict who is at risk for being high cost in the future	Claims + self-reported health status biometrics, insurance enrollment data	Employment	General health care costs	Deidentified data
Burks et al^18^	Cohort	2006–2009	Schneider National Inc.	Industry	Evaluate the effect of an employer-mandated OSA diagnosis and treatment program on non-OSA medical insurance claim costs	Claims + human resource, sleep screening data	Specific chronic condition: OSA	Specific health condition rate/costs: OSA	IRB approval
Burton et al^12^	Cohort	2009–2010	American Express	Industry	Examine associations between employee wellness program and health risks, health care costs, short-term disability absences	Claims + HRA, personnel data	Enrollment in health plan	Evaluation of employee wellness program	NR
Burton et al^19^	Cohort	2012	American Express	Industry	Examine health risks, medical conditions, and workplace economic outcomes associated with self-reported hours of sleep	Claims + HRA data	Specific chronic condition: sleep hours	General health care costs	IRB
Clark et al^20^	Cohort, longitudinal pre-/postdesign	2008–2011	Walgreens	Industry	Assess the impact on beneficiary adherence and plan sponsor cost of offering generic antidiabetic and antihyperlipidemic medications without cost sharing	Claims	Wellness program participation	Medication adherence	NR
Colombi and Wood^21^	Ecologic	2004–2007	PPG Industries	Industry	Determine the impact of population obesity on care utilization and cost of CVD conditions	Claims + HRA data	Wellness program participation	Specific health condition rate/costs: obesity and CVD	NR
Conover et al^22^	Cohort	2006–2008	SAS Institute	Industry	Examine the relationship among use of an on-site employer-provided primary care medical clinic, and health services use and health plan costs	Claims + human resource, on-site clinic data	On-site clinic use	On-site vs off-site health care costs	IRB approval + deidentified data
Cullen et al^23^	Cohort	1996–2003	Alcoa	Industry	Demonstrate that health claims data can be linked to other relevant databases such as personnel files and exposure data maintained by large employers	Claims + occupational clinic records, industrial hygiene, personnel data	Employment	Workplace injury/illness	Deidentified data
Dupree et al^24^	Cohort	2012–2017	University of Michigan	University	Investigate changes in IVF rates	Claims	Enrollment in health plan	Specific health condition rate/costs: IVF	IRB approval
Gerasimaviciute et al^25^	Cohort	2011–2013	University of Texas	University	Examine the reciprocal longitudinal associations between depression or anxiety with work-related injury	Claims + employee eligibility, workers' compensation data	Specific chronic condition: depression, anxiety	Workplace injury/illness	NR
Gibbs et al^26^	Cohort	1978–1982	Blue Cross Blue Shield of Indiana	Industry	Compare participants in the on-site health promotion programs with other employees for health care utilization	Claims + health promotion program data	Wellness program participation	Evaluation of employee wellness program	NR
Goetzel et al^27^	Cohort	2014	Lockheed Martin Corporation	Industry	Estimate the incidence, prevalence, and cost of metabolic syndrome and risk factors associated with the syndrome	Claims + biometrics, HRA data	Enrollment in health plan	Specific health condition rate/costs: metabolic syndrome	NR
Goldberg et al^2^	Cohort	2013–2017	Quest Diagnostics	Industry	Reduce the annual cost of health care without reducing access to care or adversely affecting clinical outcomes	Claims + health plan performance reports, wellness program data	Specific chronic condition: heart failure, coronary artery disease, chronic obstructive pulmonary disease, diabetes, hypertension, obesity, back and neck pain	General health care costs	NR
Gunther et al^5^	Cohort	NR	Merck	Pharma	Achieve clear improvements in the health and well-being of the workforce	Claims + biometrics, HRA, employee engagement survey data	Employment	Specific health condition rate/costs: various health conditions	NR
Hill et al^28^	Cohort	2004–2006	Arkansas state and public schools	Government	Quantify health plan costs associated with individual tobacco, obesity, and physical inactivity risks	Claims + HRA data	Enrollment in health plan	General health care costs	Deidentified data
Hillson et al^29^	Cohort	2001–2005	Centocor	Industry	Investigate the incidence, prevalence, treatment patterns, disease severity, and direct costs associated with UC	Claims	Specific chronic condition: UC	Specific health condition rate/costs: UC	IRB approval
Hincapie-Castillo et al^30^	Cohort	2015–2019	University of Florida	University	Assess the impact of the days' supply restriction from the House Bill 21 law on opioid prescribing changes	Claims	Specific prescription use	Pre- and posthealth care costs	IRB approval
Johnson et al^31^	Cohort	2004–2009	Arkansas public school and other state employees	Government	Examine PPI utilization and drug costs before and after (a) excluding esomeprazole from coverage and (b) implementing a TMAC, or reference-pricing benefit design	Claims	Specific prescription use	Pre- and posthealth care costs	NR
Khatami et al^32^	Cohort	2001–2009	University of Texas system	University	Assess the early expected benefits of waiving the copay on colonoscopy use	Claims	Enrollment in health plan	Pre- and posthealth care costs	Deidentified data
Kindermann et al^33^	Cohort	2010–2012	Cerner Corporation	Industry	Compare the influence of on-site vs off-site chiropractic care on health care utilization	Claims	Specific chronic condition: chiropractic care	On-site vs off-site health care costs	IRB approval
Leung et al^34^	Cohort	2014–2019	University of Texas	University	Characterize the longitudinal cost impact associated with a prevalent chronic illness with recurrent exacerbations	Claims	Specific chronic condition: bipolar disorder	Specific health condition rate/costs: bipolar disorder	Deidentified data
Liu et al^35^	Cohort	2004–2007	PepsiCo	Industry	Examine the impact of a comprehensive wellness program on medical costs and utilization	Claims	Wellness program participation	Evaluation of employee wellness program	NR
Maeng et al^36^	Cohort	2016–2018	University of Rochester	University	Assess the potential economic impact of a unique value-based insurance design	Claims	Wellness program participation	General health care costs	IRB approval + deidentified data
Maeng et al^37^	Cohort	2012–2015	Geisinger	Hospital system	Evaluates the program impact on care utilization and total cost of care	Claims + biometric data	Wellness program participation	Evaluation of employee wellness program	IRB approval
Merrill et al^38^	Cross-sectional	2004–2008	Salt Lake County government	Government	Evaluate the impact of the Healthy Lifestyle Incentive Program on lowering the frequency and cost of prescription drug and medical claims	Claims + HRA data	Wellness program participation	Evaluation of employee wellness program	NR
Naessens et al^39^	Cohort	January 2004	Mayo Clinic	Hospital system	Explore effects of comorbidity and prior health care utilization on choice of employee health plans with different levels of cost sharing	Claims + plan eligibility data	Employment	Employee characteristics by health plan	Deidentified data
Naydeck et al^40^	Cohort	2001–2006	Highmark, Inc.	Industry	Compare employees who participated in the program with risk-matched nonparticipants by total, annual health care expenditures, and return on investment	Claims	Wellness program participation	Evaluation of employee wellness program	NR
Nundy et al^41^	Cohort; quasi-experimental, 2-group, pre–post study	2012–2013	University of Chicago	University	Examine the impact of a 6-month mobile health demonstration project among adults with diabetes	Claims + electronic health records, text messages, phone survey data	Wellness program participation	Evaluation of employee wellness program	IRB approval
Osondu et al^42^	Cross-sectional	2014	Baptist Health South Florida	Hospital system	Examine the association of favorable cardiovascular health status with 1-year health care expenditures and resource utilization	Claims + HRA data	Employment	Specific health condition rate/costs: CVD	IRB approval
Parkinson et al^43^	Cohort	2007–2011	UPMC Health	Hospital system	Evaluate the impact of MyHealth, a wellness program, on employee health and costs	Claims + biometric, HRA data	Wellness program participation	Evaluation of employee wellness program	NR
Reeve et al^44^	Cohort, nested case-control	1993–1997	Ford Motor company	Industry	Determine the impact of exposure to metal removal fluids on workers' respiratory health, specifically NMRD	Claims	Employment	Specific health condition rate/costs: NMRD	Deidentified data
Reid et al^45^	Cohort, longitudinal pre/postdesign	2011–2013	Cerner Corporation	Industry	Measure the impact of a policy change on medication adherence, prescription fills, health care utilization, cost, and absenteeism	Claims	Specific chronic condition: anxiety, depression	Medication adherence	IRB approval + deidentified data
Rezaee et al^46^	Cohort	2008–2013	Beaumont Health System	Hospital system	Investigate MCC prevalence, and determine the relationship between MCCs and health care cost and utilization	Claims	Specific chronic condition: 20 conditions	Specific health condition rate/costs: various conditions	IRB approval
Rezaee et al^47^	Cohort	2008–2013	Beaumont Hospital System	Hospital system	Evaluate the relationship between multiple chronic conditions and urolithiasis	Claims	Specific chronic condition: 20 conditions	Specific health condition rate/costs: various conditions	IRB approval
Stenner et al^11^	Cohort	2012–2015	Vanderbilt University Medical Center	Hospital system	Examine the impact of therapeutic interchange alerts on the drug ordering behavior and the costs	Claims	Enrollment in health plan	Medication adherence	Compliance with Declaration of Helsinki on Ethical Principles for Medical Research Involving Human Subjects
Taiwo et al^48^	Case–control	1996–2009	Alcoa Inc.	Industry	Assess the relationship between acoustic neuroma and participation in a hearing conservation program and possible occupational risk factors	Claims + Human resource, injury, industrial hygiene data	Specific occupational injury	Evaluation of employee wellness program	IRB approval + deidentified data
Tollen et al^49^	Cohort	2000–2002	Humana Inc.	Industry	Determine whether the offering of a consumer-directed health plan is likely to cause risk segmentation	Claims + enrollment, employee data	Enrollment in health plan	Employee characteristics by health plan	NR
Wittayanukorn et al^50^	Cohort	2008–2010	Auburn University	University	Compare clinical and economic outcomes between patients who received and those who did not receive medication therapy management services	Claims + billing invoices	Specific chronic condition: cardiovascular conditions	Pre- and posthealth care costs	IRB approval

^a^ Exposure: condition for entry into the study, for example, employment within a company, presence of a disease, or a workplace intervention; outcome: evaluated endpoints, for example, health care costs, prevalence of a condition, rate of workplace injury.

CVD, cardiovascular disease; HRA, health risk appraisal; IRB, institutional review board; IVF, *in vitro* fertilization; MCC, multiple chronic condition; NMRD, nonmalignant respiratory disease; NR, not reported; OSA, obstructive sleep apnea; PPI, proton pump inhibitor; TMAC, therapeutic maximum allowable cost; UC, ulcerative colitis; UPMC, University of Pittsburgh Medical Center.

Objectives of the included studies ranged from understanding the existing employee health status to targeting a specific health condition and implementation of wellness programs. Of the 41 studies, 24 (59%) supplemented claims data with various other data sources, including biometrics, on-site clinic records, worker's compensation records, industrial hygiene exposure data, and human resource data to achieve study objectives.

### Study exposures and outcomes

**FIG. 2.**
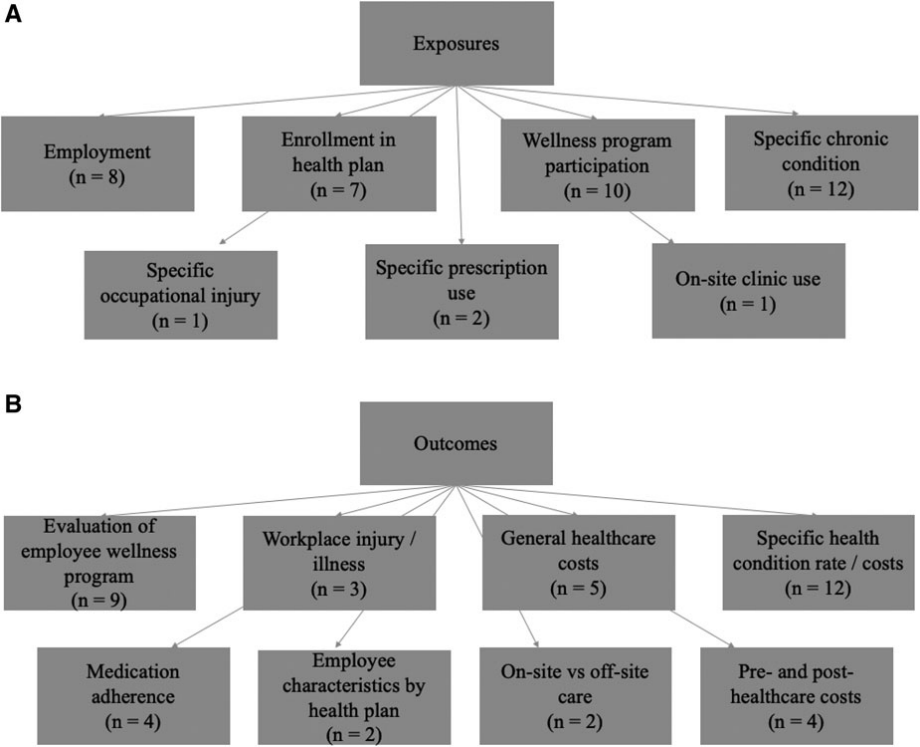
**(A)** Exposures examined in the included studies. **(B)** Outcomes examined in the included studies.

A variety of exposures and outcomes were examined in the included studies, shown in Figure 2A and B, respectively. The most common exposures were specific chronic conditions (*n* = 12; 29%), participation in a company wellness program (*n* = 10; 24%), and employment within the company (*n* = 8; 20%). Other exposures included enrollment in a health plan (*n* = 7; 17%), specific occupational injury (*n* = 1; 2%), specific prescription use (*n* = 2; 5%), and on-site clinic use (*n* = 1; 2%). The most common evaluated outcomes include the rate and costs associated with a specific health condition (*n* = 12; 29%), effectiveness of employee wellness programs (*n* = 9; 22%), and general health care costs (*n* = 5; 12%).

Additional outcomes included health care costs pre- and postintervention (*n* = 4; 10%), medication adherence (*n* = 4; 10%), workplace injury/illness (*n* = 3; 7%), on-site versus off-site care (*n* = 2; 5%), and employee characteristics by health plan (*n* = 2; 5%). Refer to Table 2 for the specific chronic and health conditions considered in these studies.

### Measures to protect patient confidentiality and privacy

Of the 41 studies, 12 (29%) reported that the study was approved by an institutional review board (IRB) while 7 (17%) indicated that the authors received deidentified data for analysis (Table 2). Five (12%) studies noted both an IRB approval and receipt of deidentified data. Compliance with the Declaration of Helsinki on Ethical Principles for Medical Research Involving Human Subjects was reported in 1 study (2%).^11^ Sixteen studies (39%) did not report on how confidentiality and privacy were maintained throughout study conduct.

## Discussion

This ScR reviewed 41 studies published between 1985 and 2022 that described the ways in which self-insured employers have examined their employee health claims data in the United States. The majority were cohort studies conducted by industry employers and supplemented claims data with other data sources such as industrial hygiene data. A variety of exposures, such as specific prescription use or participation in a wellness program, and outcomes, such as cost-effectiveness of an on-site clinic or the rate and cost of a specific health condition, were examined in the studies.

The authors of the included studies identified several lessons learned from conducting these analyses of the employee health claims data, including both global themes pertaining to the health of the entire workforce and more program-specific conclusions. Several studies concluded that adequate and continued investments are needed to build a culture of wellness. Others stated that diverse data sets are essential for understanding the current health status of employees and identify gaps for improvement within the organization.

One study indicated that it is imperative that preventive and disease management services are offered so that low-risk employees may remain in the low-risk group while high-risk employees may achieve better health over time through utilization of those services. Studies that evaluated program-specific objectives concluded that on-site health services, especially employer-sponsored pharmacies, may reduce medication adherence barriers, and savings from on-site clinics are dependent on the cost of care compared with avoided claim costs.

Another study reported that worker's compensation costs were found to decline with the use of a small network of health care providers who maintained effective communication between them. Medication therapy management services were found to be a significant factor in achieving health goals and improving disease states as well as reduction of pharmacy, medical, and overall expenditures in several studies. Lastly, as the workforce age, distribution shifts to younger workers, 1 study concluded that employers will need to address a growing demand for mental health services.

Strengths of the included studies and analyses using claims data include the diverse study populations and outcomes evaluated across studies. The employer-led analyses were able to draw on large amounts of data covering historical and current information on health care utilization, rates of diseases and conditions, and costs to both employee and employer. Moreover, these large claims data sets were able to be linked with smaller sources such as industrial hygiene and workers' compensation data to add valuable information to the analyses not available from claims data. Several limitations were also identified by the study authors.

This review only identified 41 studies that may reflect publication bias; many self-insured companies have access to claims data and may conduct analyses but for reasons unknown, not many studies are published. The populations are often self-selected, such as voluntary participation in wellness programs or use of on-site care, which may limit the generalizability of the results. Majority of studies were from industry with limited representation from government entities. Use of self-reported data such as health risk assessments to supplement the claims data could bias the outcomes since not all individuals self-report health information accurately due to poor recall, potential stigma regarding certain conditions, or fear of recourse by the employer.

There is also the potential for underestimation of adverse health outcomes because of the healthy worker effect (ie, active employees tend to be healthier than the unemployed) and the fact that some outcomes, such as obesity, may not be able to be captured appropriately in claims data. Lastly, costs may be underestimated since most of these studies did not consider administration costs of programs or policy changes.

When examining claims data, it is critical to protect patient and employee privacy. A strategic collaboration between employees and employers as they determine what programs are needed could help gain employee buy-in into these changes and motivate them to participate in additional data collection methods. As seen in many of the included studies, other sources of data were utilized to supplement information gained from claims. For example, 1 employer combined claims data with health risk appraisal (HRA) and company personnel data to evaluate the effectiveness of their new wellness program.^12^ The addition of HRA data increased awareness of employee's health status, which, in turn, increased participation in the program. Employer investment in such a program lowered health costs and improved productivity.

Many studies were excluded because they were initiated by third parties such as pharmaceutical companies to assess the effectiveness of a drug/s, etc. One excluded study that evaluated the cost of asthma to an employer was supported by a grant from Immunex, a biotech company that developed asthma and other treatments, and conducted by several research groups.^13^ A limitation noted by the authors was the lack of information on lifestyle factors and comorbid conditions that could impact asthma severity and related costs. Short-term medical-related absences without claims and reduced productivity when ill employees remained at work were costs to employers that were not captured in analyses.

Had the employer been involved and included buy-in from employees, this information may have been able to be collected for a more robust analysis of asthma and impact on its employees. Strategic investments in employee health not only benefit the employees but also improve productivity, job satisfaction, and could result in savings for employers.

This ScR had several strengths, including strong study methodology involving *a priori* registration of the study protocol, adherence to the PRISMA-ScR framework, and inclusion of multiple exposures and outcomes of employee populations with self-insuring employers. Since the ScR was specific to the United States and self-insured employee populations, findings may not be generalizable to those outside of the United States or companies that are not self-insured. Furthermore, the review was unable to account for changes in measures to maintain patient privacy and reporting guidelines over time.

Self-insured employers across varied sectors have used employee health claims data to gain insights into their employees' health, well-being, productivity, and health care utilization. These data offer the ability to investigate a range of objectives since multiple exposures and outcomes can be assessed simultaneously with continued follow-up. Once gaps are identified, different departments across the organizations can join to determine the efforts required to build a culture of wellness. Employee health claims data offer a unique opportunity for employers to yield additional savings in the long run through implementation of wellness and other targeted programs and have a lasting, positive impact on their employees.
